# Expression of xylosyltransferases I and II and their role in the pathogenesis of arthrofibrosis

**DOI:** 10.1186/s13018-020-1544-8

**Published:** 2020-01-23

**Authors:** Anke Bernstein, Sven N. A. Reichert, Norbert P. Südkamp, Sergio Latorre Hernandez, Andreas G. Nerlich, Jan Kühle, Hermann O. Mayr

**Affiliations:** 1 G.E.R.N. Tissue Replacement, Regeneration & Neogenesis, Department of Orthopedics and Trauma Surgery, Medical Center - Albert-Ludwigs-University of Freiburg, Faculty of Medicine, Albert-Ludwigs-University of Freiburg, Germany, Hugstetter Straße 55, 79106 Freiburg, Germany; 2 Department of Orthopedics and Trauma Surgery, Medical Center - Albert-Ludwigs-University of Freiburg, Faculty of Medicine, Albert-Ludwigs-University of Freiburg, Germany, Hugstetter Straße 55, 79106 Freiburg, Germany; 3 Institute of Pathology, Academic Clinics München-Bogenhausen and München-Schwabing, 81925 Munich, Germany; 4 Department of Knee, Hip and Shoulder Surgery, Schoen Clinic Munich Harlaching, Harlachinger Strasse 51, 81547 Munich, Germany

**Keywords:** Xylosyltransferase, Arthrofibrosis, Pathogenesis, Myofibroblast, Immunohistology

## Abstract

**Background:**

Arthrofibrosis is a painful and restraining complication that occurs after about 10% of total knee arthroplasty and cruciate ligament surgery. The pathogenesis of arthrofibrosis has not yet been fully understood. Stress signals stimulate immune cells, and fibroblast differentiates into myofibroblast, which produce a large amount of collagen. Xylosyltransferases also appear to be involved in these pathways. They catalyze proteoglycan biosynthesis, which is involved in tissue remodeling and myofibroblast differentiation. The aim of this study was to investigate the relationship between the disease arthrofibrosis and the expression of the two isoforms of xylosyltransferases I and II.

**Methods:**

Tissue samples from 14 patients with arthrofibrosis were compared with tissue samples from seven healthy controls. The xylosyltransferases were detected by immunohistochemistry. The tissues were divided into four different areas of interest: vessels, synovialis, cell-poor and cell-rich fibrosis, or cell-poor and cell-rich areas in the control group. A quantification of the results was performed by modification of the immunoreactive score according to Remmele and Stegner.

**Results:**

Xylosyltransferase I was expressed in the various tissue types at varying rates. Xylosyltransferase I expression was considerably and significantly stronger than that of xylosyltransferase II. The following sequences of xylosyltransferase I and xylosyltransferase II expression were determined as follows: vessels >> cell-rich fibrosis > cell-poor fibrosis > synovialis. A positive correlation between the number of positive fibroblasts and the immunoreactive scoring system (IRS) was documented.

**Conclusions:**

The significant positive correlation of xylosyltransferase -I expression with increasing number of fibroblasts demonstrates a high myofibroblast differentiation rate, which implies a gradual event as the pathogenesis of arthrofibrosis.

## Background

Arthrofibrosis (AF) is a severe complication after trauma and surgical interventions in joints. AF is understood as the extensive inflammatory growth of connective tissue resulting from overshooting fibroblast proliferation in the knee joint leading to restricted mobility and often pain. It occurs following trauma, surgery, or an infection, as well as after joint arthroplasty, although one must distinguish it from other diseases whose clinical presentation is similar [1–3]. This means that the proven limited range of motion of the knee is not due to misplacement of grafts or implants or bony structures in ligament reconstruction, but is due to infection or pain due to chronic regional pain syndrome (CPRS) [4]. Characteristic of AF is fibrotic thickening of the joint capsule that when most severe can cause the total loss of joint mobility [5–7]. Its incidence is reported to lie between 4 and 35% after tendon surgery and endoprostheses in the knee joint [8–11]. Two clinical etiological forms of AF are known: primary and generalized or secondary and localized. Primary AF is characterized by general increase of connective tissue within the entire joint and the periarticular tissue leading to limitation in joint mobility. There is no specific reason for primary AF. Secondary AF is usually caused by local, mechanical problems or infection [12]. AF is categorized histopathologically in the joint arthroplasty-associated form, the diffuse, non-joint arthroplasty-associated form, and the local, non-joint arthroplasty-associated form.

Cytokines like TGF-β1 (transforming growth factor β1) and PDGF (platelet-derived growth factor) play essential roles in the development of AF [13]. These cytokines are inducing proliferation and differentiation of resident fibroblasts to active myofibroblasts, which are modified fibroblasts that are able to contract [14]. Wound healing is physiologically controlled by the apoptosis of myofibroblasts. If such apoptosis fails to occur, extracellular substances continue to be secreted, followed by a pathological buildup of scar tissue through tissue contraction and an eventual loss of mobility [10, 15]. The inflammatory cytokines and mediators that trigger fibrosis are essential components of a healthy immune system. Typically, inflammatory cytokines are downregulated after some time. However, they are still present, which can lead to the development of a fibrosis. The appearance of an inflammatory cytokine causes the formation of receptors for other cytokines and sensitizes the cells to react [16]. They are also characterized by their high rate of synthesizing components of the extracellular matrix (ECM) like collagens and xylosyltransferases (XT). XTs catalyze the speed-determining step known as glycosaminoglycan biosynthesis and play a major role in forming the ECM. A rise in xylosyltransferase I (XT-I) activity furthers the accumulation of proteoglycan characteristic of fibrotic diseases [17, 18]. A trial that enrolled 95 patients with AF and 132 controls, after total knee arthroplasty (TKA), reported no significant XT-I activity in serum [10]. However, patients with other systemic fibrotic diseases like systemic sclerodermia or liver fibrosis did demonstrate elevated serum-XT activity compared to a control cohort [17, 19]. The cause thereof may be a local circumscribed process through the synovial membrane that cannot be clinically proven via systemic parameters like increased serum-XT activity. To the best of our knowledge, there has been to date no immunohistological analysis of XT (XT-I and XT-II) expression in human arthrofibrotic tissues. The primary aim of the current research was to analyze the expression of and to localize both isoforms in arthrofibrotic knee joint biopsies.

## Materials and methods

This study was approved by the institutional review board of the authors’ institution (institutional review board no. 305/10 and 610/14).

### Patients

Tissue samples from knee joints of 14 patients (3 female, 11 male, average age 38 years) in this study treated in the same hospital between 2004 and 2015 were examined. They suffered from anterior cruciate ligament (ACL) rupture and were mostly treated by ACL reconstruction (Table 1). The selected patients developed arthrofibrosis diagnosed according to clinical and arthroscopic criteria. There is no definitive diagnostic imaging test available for diagnosing post-surgical knee fibrosis. Knees were investigated by plain radiographs as well as CT imaging. There is currently not enough evidence for the routine use of MRI in diagnosing fibrosis [4]. Arthroscopic arthrolysis was performed as therapy. The fibrotic tissue was excised.

**Table 1:** Summary of patient/procedure information relating biopsies

Type of intervention	Patient numbers
ACL reconstruction	6
ACL reconstruction and meniscus refixation	2
ACL rupture without intervention	2
ACL reconstruction following knee joint infection	1
Osteosynthesis of a tibial head fracture and posterior cruciate ligament (PCL) reconstruction	1
Implantation of TKA	2

The control group consisted of seven postmortem knee joint tissue specimens provided by the Anatomical Institute Basel originating from three women and three men. Their mean age was 86 years. The work group was unaware of any knee disease among the donors.

### Immunohistological staining

Tissues were stored in 4% formalin fixed and then embedded in paraffin. Slices of 5-μm thick were routinely stained with Giemsa and hematoxylin/eosin (HE) to determine the grade of AF and measure β-catenin expression. For β-catenin staining (BD Biosciences, 1:250), specimens were pretreated for 15 min in the microwave at 99 °C in boiling-hot citrate buffer (Dako REAL Target Retrieval Solution) at pH 6.

For immunostaining, ZytoChem Plus HRP Polymer Kit (Zytomed Systems GmbH, Berlin, Germany) was used.

Sections were first incubated with primary antibodies overnight at 4 °C after blocking endogenous peroxidase with 3% hydrogen peroxide (peroxide block) for 10 min. All antibodies were diluted 1:500 to enable optimal staining results. Background staining caused by unspecific binding of the primary or secondary antibody in the HRP polymer is minimized by incubating in a protein-blocking solution (5 min).

Sections were then washed with wash buffer. After washing, enhancement reagent (“PostBlock“) was applied (20 min) and incubated. A second washing was followed by applying the HRP polymer (30 min). Any excess of unbound HRP polymer is thoroughly washed away after incubation and incubated with biotinylated goat anti-rabbit antibody for 30 min at 37 °C, followed by incubation for 30 min in streptavidin-biotin-peroxidase complex. After rinsing in phosphate-buffered saline (PBS) and Tris buffer, color reaction was developed with 3-amino-9-ethylcarbazol substrate, followed by counterstaining with HE. Localized red-brown staining characterized a positive reaction. As negative controls, the primary antibodies were replaced by PBS. Tissue samples obtained from six knee cadavers without any macroscopic pathology in the synovial tissue served as control. Polyclonal antibodies (MyBioSource, San Diego, USA) were used to immunohistologically localize XT-I and XT-II. Here, the HPF (high-power field) in the present paper was defined as an area of 34.366 μm^2^.

For immunohistological differentiation of fibroblasts and myofibroblasts, an antibody against α-smooth muscle actin (Firma Abcam, diluted to 1:62.5) was used. To monitor whether the fibroblasts were β-catenin-positive cells with fibroblast-like morphology (and were actually fibroblasts and not histiocytes or macrophages), we stained the specimens with CD68 (DakoCytomation, 1:20).

### Histological grades of AF

Histopathological graduation of fibrosis in AF [20] was determined by more exact, semi-quantitative criteria, so-called fibroblast cellularity (the fibroblasts’ distances from each other) (Table 2).

**Table 2:** Histopathological graduation of fibrosis in AF

Grade 1	
Low-grade fibroblastic cellularity; distance between fibroblasts is more than 2 cell lengths (of fibroblasts).	
Grade 2	
Medium-grade fibroblastic cellularity; distance between 2 fibroblasts is less than 2 cell lengths (of fibroblasts).	
Grade 3	
High-grade fibroblastic cellularity; the distance between fibroblasts is less than one cell length (of fibroblasts); cytoplasm and nuclei of fibroblasts may be in contact.	

### Immunohistological analysis

Specimens were histologically analyzed under an optical microscope from Olympus (BX51). The Stream Motion 1.9.4 software from Olympus Soft Imaging Solutions GmbH was used for image analysis.

The AF group’s tissues we examined were classified in four categories: vascular tissue (V), synovial tissue (S), hypocellular fibrosis (HYP-F), and hypercellular fibrosis (HYPR-F).

The control group’s tissues were categorized in V, HYPR, HYP, or S, as no fibrosis was present.

Fibrosis was classified as hypercellular if the total cell count exceeded 25 cells and as hypocellular if the total cell count was between 5 and 25; the ratio between the hypercellular tissue’s total cell count and that of the hypocellular tissue had to exceed 2.5 in consultation with Prof. A. Nerlich (MD, Pathologist (Municipal Hospital Munich, Institute of Pathology). The total cell count corresponded to the mean of cell counts from 12 different regions of interest (ROIs). The ROI measured 34.37 μm^2^.

To assess the expression of XTs, staining in each specimen was evaluated in three ROIs per tissue type. As for region of interest (ROI), we determined a vision field at × 40 magnifications. To validate measurements, immunohistochemical staining was carried out twice with each antibody, resulting in six ROIs per specimen. These were scored according to an immunoreactive scoring system (IRS [21]) that enables semiquantitative analysis of the staining. Here, the number of cells staining positive is multiplied by the stain’s intensity, yielding values totaling between 0 and 12. The numbers of positive cells were allocated in line with recommendations from Prof. Nerlich (MD, Pathologist, Municipal Hospital Munich, Institute of Pathology) in grades 0 to 4 (Table 3).

**Table 3:** Immunoreactive score (Remmele and Stegner) after modification in consultative cooperation with Prof. Dr. A. Nerlich, MD, Pathologist (Municipal Hospital Munich, Institute of Pathology)

Color intensity	No stain reaction	Weak stain reaction	Moderate stain reaction	Strong stain reaction
Positive cells	0 points	0 points	0 points	0 points
No positive cells, 0 points	IRS = 0	IRS = 0	IRS = 0	IRS = 0
< 10% positive cells, 1 point	IRS = 0	IRS = 1	IRS = 2	IRS = 3
10–25% positive cells, 2 points	IRS = 0	IRS = 2	IRS = 4	IRS = 6
26–50% points positive cells, 3 points	IRS = 0	IRS = 3	IRS = 6	IRS = 9
51–100% positive cells, 4 points	IRS = 0	IRS = 4	IRS = 8	IRS = 12

### Statistical assessments

For statistical assessment, Statistical Analysis Software (SPSS) from IBM, version 23, was used. To determine differences in the expression of xylosyltransferase between AF patients and our controls, we calculated the medians of the six ROIs per specimen. To assess a specimen’s total staining, the means of the expression of all four tissue types were included and the aggregate value was calculated. The Mann-Whitney *U* test (nonparametric test for two independent, not normally distributed variables) facilitated the statistical analysis. The considered statistically significant were *p* values < 0.05. Correlations between the time interval index-OP until tissue removal and XT expression, as well as between the expression of other immunohistochemical markers (β-catenin and CD-68) and XT expression, were determined according to Spearman‘s rank correlation analysis.

## Results

### Controls

**Fig. 1:**
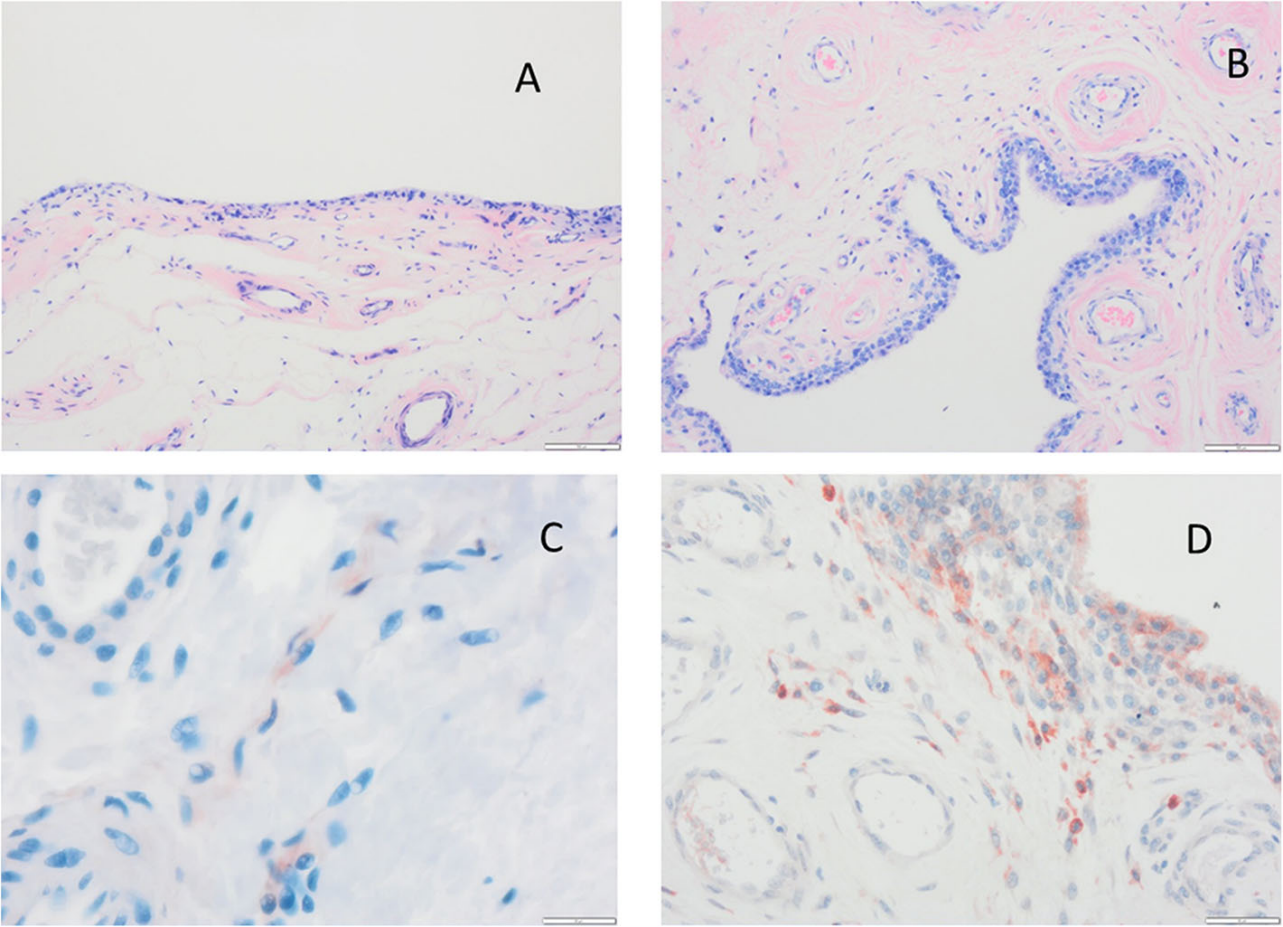
HE Staining of soft tissue. **a** Synovial tissue from the control group. Intima has one to three strata, loose connective tissue, superficial network of capillaries connected to the intima. **b** AF tissue from a patient. Synovial hyperplasia, enlarged subintima with vascular infiltration, fibrosis. **c** β catenin expression in a patient’s AF tissue. **d** AF tissue exhibits macrophages in the intima with typical immunohistological staining for CD68 in close vicinity to vessels. AF arthrofibrosis

The control group’s tissue specimens revealed histologically a single-stratum to three-strata synovial intima connected by a loose connective tissue with a few narrow vessels (depending on the wall thickness) (Fig. 1a) [22, 23].

### Arthrofibrosis

The patients with AF revealed obvious intimal hyperplasia in the synovia with more than three cell layers (Fig. 1b). The adjacent subintima is fibrotic, as evident through increased presence of fibroblasts and fibrocytes. In addition to the subintimas being hypervascular, the vessel walls are morphologically too thick (Fig. 1b). Areas of manifest fibrosis have become attached to the fibrotic subintima with no signs of vascularization. Immune staining with β-catenin displays different numbers of β-catenin-positive fibroblasts (Fig. 1c).

### Expression of xylosyltransferase I in AF and controls

**Fig. 2:**
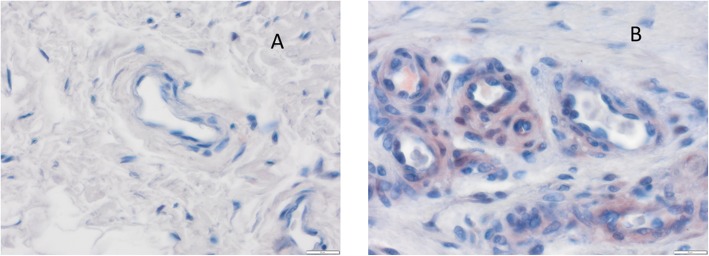
Immunostaining of vessels with xylosyltransferase I. **a** Control. **b** Typical specimen from an arthrofibrosis (AF) patient

XT-I was expressed in the various tissue types at varying rates. The cytoplasm of the cells was obviously stained (Fig. 2). Specimens from each tissue type revealed cells stained to different regional degrees of intensity, i.e., some vessels or parts of the synovia showed marked color intensity, while others were less intensely stained.

Vessels from AF patient’s specimens displayed the strongest staining for XT-I. Thickness of these vessel walls were obviously and morphologically excessive compared to the control groups. Manifest fibrosis attached to the fibrotic subintima with no apparent vascularization.

**Fig. 3:**
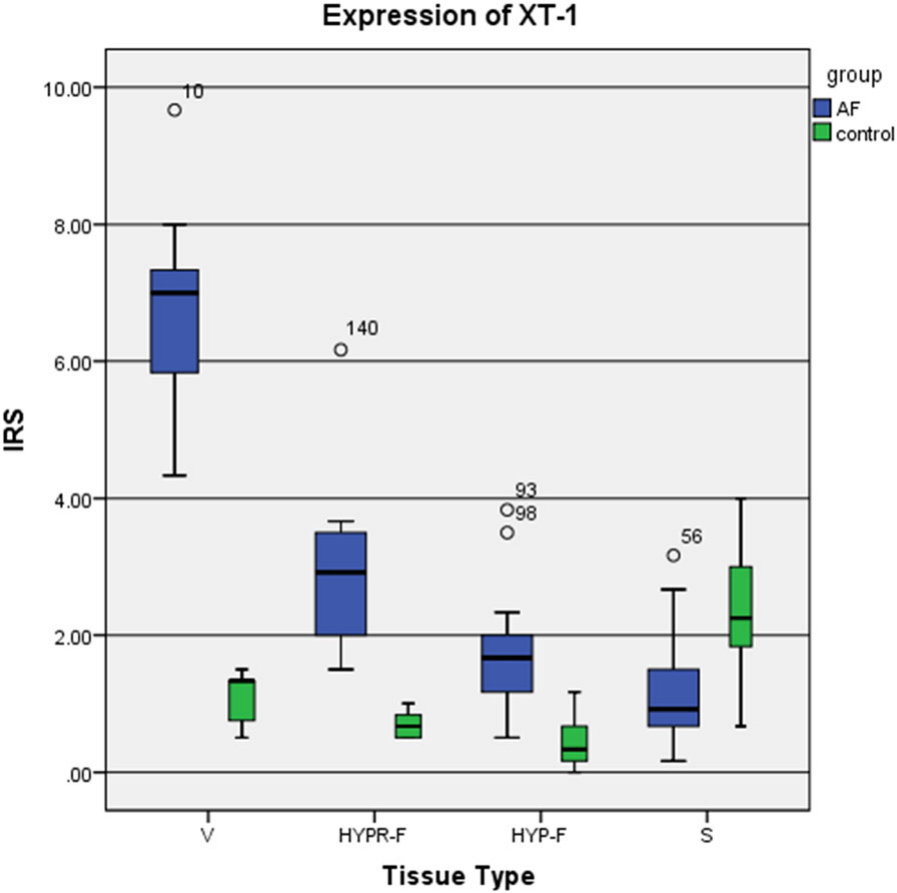
Immunoreactive scoring system (IRS) of xylosyltransferase I (XT-I) for each type of tissue. Statistical significance (Table 4)

In addition to cytoplasm staining with perinuclear accentuation, some of the intercellular spaces’ connective tissue also stained positive for XT-I. The vascular intima’s cells stained positive in 10 of 14 specimens (> 50% of the total cell count); in the remaining specimens, 4 of 14 stained positive (25% and 50%). Staining intensity was in ten specimens moderate, in two strong, and in another two weak. The median immunoreactive score for XT-I according to Remmele and Stegner was 7.00 in the AF group and 1.33 in the control cohort (Fig. 3).

Every specimen from the AF group exhibited greater stain intensity than the control groups—a significant difference (Table 4). A positive linear correlation between the number of fibroblasts staining positive and the IRS (correlation coefficient after Cohen *r* = 0.81) was observed. In the areas surrounding hypercellular fibrosis, the AF specimens’ fibrocytes and fibroblasts (IRS = 2.95) were observed to be more intensively stained than those of the control group (IRS = 0.67). XT-I expression was significantly stronger (*p* < 0.001, Table 4). In particular, the staining was perinuclear, and some of the cell processes were intensively stained. All the AF group’s specimens were more intensively stained than the control groups. Here too, a strong positive correlation between the number of positive fibroblasts and the IRS was noted (*p* < 0.001 bilateral, correlation coefficient after Cohen 0.800).

**Table 4:** Statistical significance—IRS

	Vessel	HYPR-F	HYP-F	Synovium
AF group versus control				
XT-1	*p* < 0.001	*p* < 0.001	*p* = 0.001	*p* = 0.075
XT-2	*p* = 0.001	*p* = 0.002	*p* = 0.154	*p* = 0.175
XT-1 versus XT-2				
AF group	*p* < 0.001	*p* = 0.001	*p* = 0.006	*p* = 0.007
Control group	*p* = 0.026	*p* = 0.001	*p* = 0.230	*p* = 0.003

Hypocellular fibrosis revealed a weaker stain than hypercellular fibrosis. In most of the cases, the proportion of positively stained cells was 10 to 25%. Stain intensity was largely graded as weak. Staining was primarily perinuclear. The connective tissue exhibited staining rarely. The AF patients’ IRS median equaled 1.67, the control group’s 0.33—a significant difference (*p* < 0.001). Here, also, a positive correlation between the number of positive fibroblasts and the IRS was observed (effect strength after Cohen *r* = 0.71).

The control’s synovial membrane stained more strongly (median 2.25) than that of the AF patients (median 0.92). Stain was limited to the extensive cytoplasm of the synoviocytes. Difference was not significant.

**Fig. 4:**
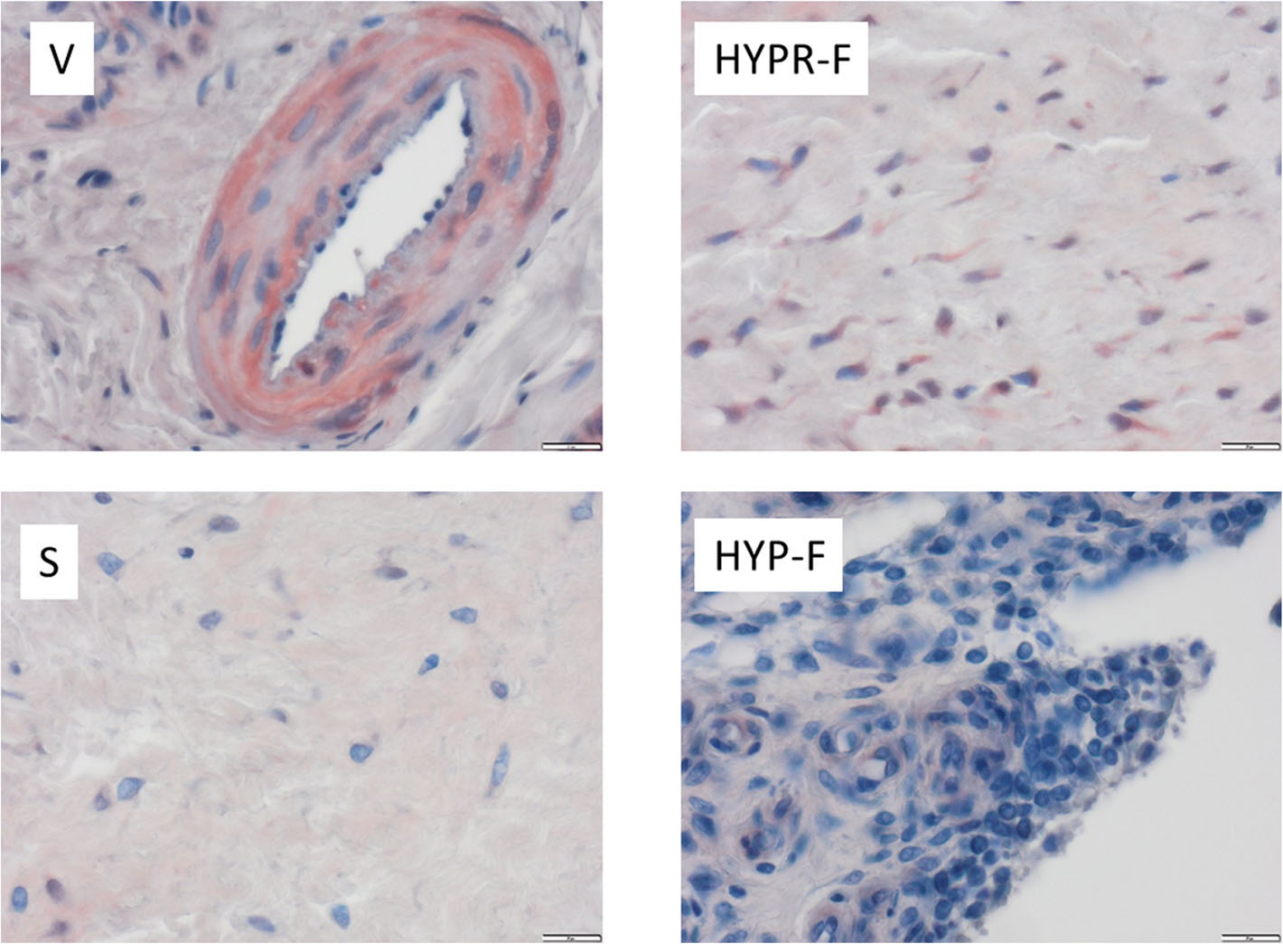
Expression of xylosyltransferase I (XT-I) according to tissue type (vascular tissue (V), synovial tissue (S), hypocellular fibrosis (HYP-F), and hypercellular fibrosis (HYPR-F))

Comparison of the four tissue types (V, HYPR-F, HYP-F, and S) revealed that XT-I expression was strongest in the vessels (Fig. 4, Table 4).

The sequence below shows the order of XT-I expression strength:

$$ V\kern0.36em >> HYPR-F\kern0.36em >\kern0.36em HYP-F>S $$

Considering all the tissue types together, one notes that the AF group’s specimens were stained with the exception of synovia significantly stronger than the control’s (Table 4) and that XT-1 expression thus approximately tripled. The IRS median (V, HYPR-F, HYP-F, S) of both groups were compared: while the AF group’s median of expression equaled 3.00, the control’s was 1.04.

### Xylosyltransferase II expression in AF and controls

**Fig. 5:**
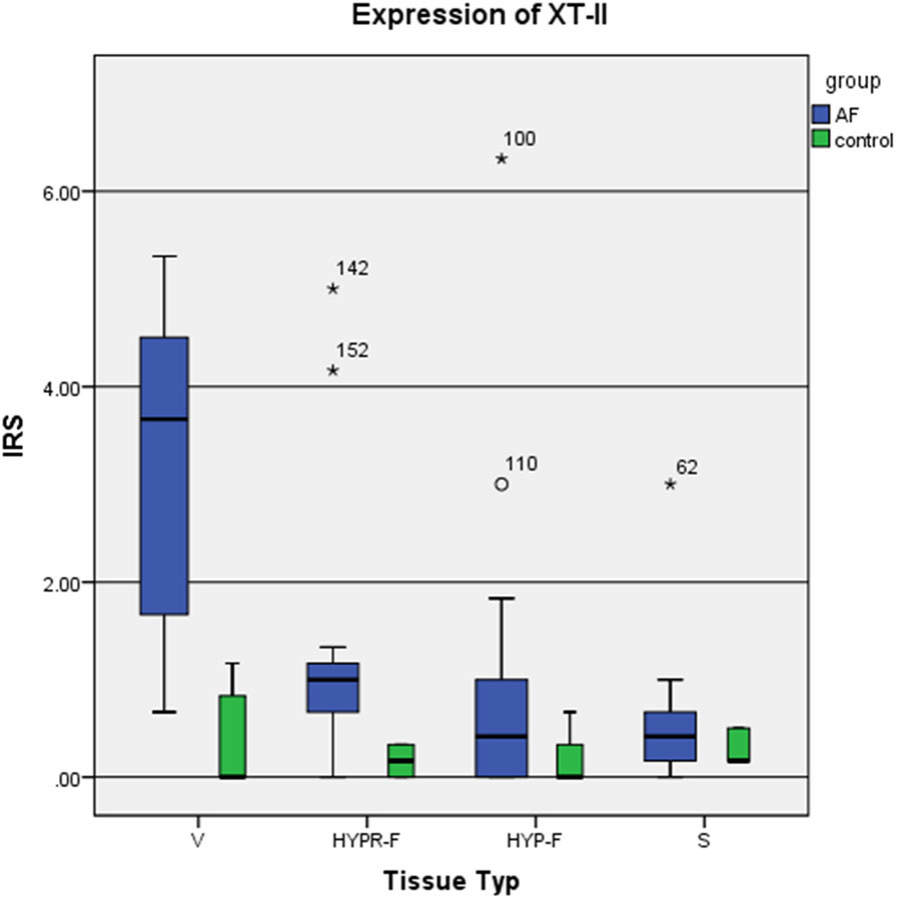
Immunoreactive scoring system (IRS) of xylosyltransferase II according to tissue type. Statistical significance (Table 4)

XT-II expression proved to be variously strong in the four tissue types (Fig. 5, Table 4). Here, too, stronger- and weaker-stained cells in the given specimen were observed.

**Fig. 6:**
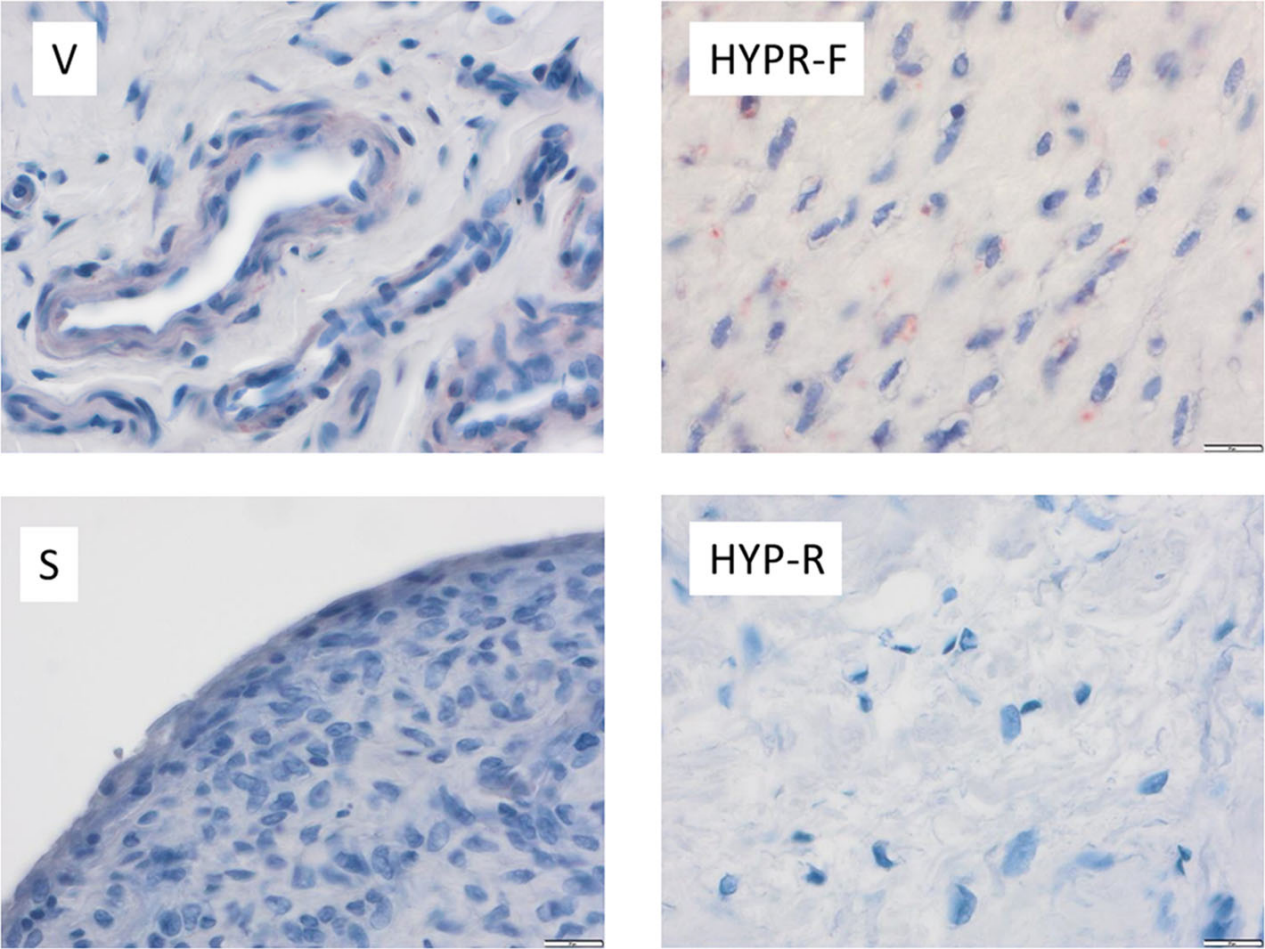
Expression of xylosyltransferase II (XT-II) according to tissue type (vascular tissue (V), synovial tissue (S), hypocellular fibrosis (HYP-F), and hypercellular fibrosis (HYPR-F))

Vessel cells stained most strongly (V, Figs. 5 and 6, Table 4). Nearly all the AF group’s specimens stained more intensively than the control’s. In most cases (10 of 14), the percentage of stained cells was between 25 and 50%. The stain intensity ranged from weak to medium strong. The IRS median in the AF group was 3.67, the control’s 0.00. The difference between the two groups in staining intensity was with the exception of HYP-F and synovia statistically significant (Table 4), and there was a linear correlation between the number of positively stained fibroblasts and the IRS (correlation coefficient after Cohen *r* = 0.76).

The AF group’s staining of cells in hypercellular fibrosis (HYPR-F, IRS = 1.0) was stronger than the control’s (IRS = 0.17). We observed cytoplasm stained with perinuclear accentuation. The cell processes of fibrocytes and fibroblasts were not stained. Up to 25% of the majority of cells in the specimens was stained; intensity of stain was graded as weak. This difference was significant (*p* < 0.001). Here, we also observed a positive correlation between the number of positive fibroblasts and the IRS (effect strength after Cohen *r* = 0.67).

Hypocellular fibrosis (HYP-F), however, stained more weakly for XT-II: very few cells were stained. The IRS equaled 0.42 for the AF patients and 0.00 for the control. Difference was not significant.

Cells in the synovial membrane (S) also stained weakly. Compared to the control, the synoviocytes from the AF group’s specimens stained more strongly, their IRS median equaling 0.42, whereas the control’s was 0.17. The staining difference here was not significant either.

Comparing the four tissue types V, HYPR-F, HYP-F, and S, it was observed that XT-II expression was strongest in the vessels (Fig. 4, Table 4).

The sequence below shows the order of XT-II expression strength:

$$ V\kern0.36em >>\kern0.36em HYPR-F\kern0.36em > HYP-F>S $$

Considering all the tissue types together, one notes that the AF group’s specimens were stained almost significantly stronger than the control’s (Table 4) and that XT-II expression was thus considerably stronger. The IRS medians of XT-II expression (V, HYPR-F, HYP-F, S) of both groups were compared: while the AF group’s median of expression was 1.46, the control’s equaled 0.27—a significant difference (*p* < 0.001).

## Discussion

In the present research, expression of XT-I and XT-II in arthrofibrotic biopsies of knee joints was analyzed. It was categorized by tissue type: vascular tissue, synovial tissue, hypercellular fibrosis, and hypocellular fibrosis.

XT-I and XT-II were expressed differently in the various types of tissues. AF group’s specimens were stained almost significantly stronger than the control’s. XT-I expression was considerably stronger than that of XT-II. The difference was with exception of synovia significant. Vascular tissue exhibited the strongest expression, the synovialis the weakest. A positive correlation between the number of positive fibroblasts and the IRS was documented.

Arthrofibrosis, a consequence of a pathological increase in connective tissue, causes restricted mobility in the affected joint [1, 24]. Wound healing begins after a surgical intervention, culminating in scar formation. This healing process is dynamic and multilayered.

Dissemination of pro- and anti-inflammatory mediators from thrombocytes, damaged tissue, and recruited, infiltrating cells from the immune system leads to activation and differentiation of fibroblasts. Myofibroblasts are created [15, 25]—an intermediate form of connective-tissue cells and smooth muscle cells with the ability to contract and are capable of storing the extracellular matrix: ECM—proteins like collagen and fibronectin are capable of synthesizing XT [10]. The apoptosis of myofibroblasts occurs at the end of the physiological healing process. Fibrotic anomalies are usually characterized by abnormally high myofibroblast formation. Identifying the cellular sources thereof is clinically relevant, as they pave the way to better understanding the pathological mechanisms in fibrosis development on the cellular level and thus also the potential means of developing selective anti-fibrotic agents for effective therapy [25].

Arthrofibrosis affects people of all ages, although it is rare in children [16]. The number of myofibroblasts in the tissue from arthrofibrotic knees can be ten times higher than in healthy subjects [26]. Xylosyltransferases catalyze the production of proteoglycans associated with fibrosis and are involved in tissue remodeling and myofibroblast proliferation [26]. There are only few studies concerning the influence of the age of the patients on the expression of xylosyltransferases. Prante et al. investigated the XT-I and XT-II expression in cardiac fibroblasts and in patients with dilated cardiomyopathy and compared findings with nonfailing donor hearts. No significant changes were observed for XT-II mRNA expression. For both groups, no age-related changes in the XT-I expression could be detected [18].

It is generally assumed that the sources of myofibroblast populations in fibrotic diseases are considerably heterogeneous. Myofibroblasts can be derived from resident fibroblasts, pericytes, and bone marrow (BM) cells. The latest literature indicates that resident perivascular MSC-like cells as well as MSC-like pericytes and fibroblasts are the main sources needed to develop kidney myofibroblasts [25, 27–30]. There is also evidence that endothelial and epithelial cells cannot differentiate to form myofibroblasts [25, 31–33]. Growth factor TGF-β1 (transforming growth factor β1) is considered a master regulator in the differentiation of myofibroblasts [25]. It induces the synthesis of collagens, proteoglycans, and other ECM molecules in the target cells. TGF-β1 can also induce the production of XT-I in fibroblasts [10, 17]. XT-I and XT-II catalyze the posttranslational xylosylization of proteoglycan *core* proteins in the Golgi complex [34]. The O-glycosidic transfer of a xylose molecule onto specific serine deposits occurs via the conversion in the UDP-xylose substrate. Although xylosylization takes place in the Golgi complex, more than 90% of the xylosyltransferase in cell culture experiments is not detected in the cells, but rather in the cell culture medium [17, 35]. Only a small proportion of total XT activity is found to be membrane-bound [35]. XT’s secretion into the extracellular interstitial space co-occurs with large proteoglycans [34]. XT-I and XT-II are expressed differently in various types of cells and tissues. XT activity has been proven in cell culture medium of various cell lines [17, 36, 37] and in many human body fluids like blood, synovial fluid, follicular fluid, and cerebrospinal fluid [10, 17, 34–36, 38–41].

It is known that AF development is encouraged by both TGF-β1 and mechanical stress acting on the synovial membrane. Synovial fibroblasts are subjected to particular stress in the knee joint that occurs via stretching and the body’s weight, as well as shearing effects of the synovial fluid’s movement [42, 43].

There is evidence that XT also plays a role in fibrosis development. XT is strongly expressed in the lung tissue of bleomycin-induced pulmonary fibrosis in rats and in heart muscle biopsies from patients with dilative cardiomyopathy [34].

To the knowledge of the work group, no tissue has been proven to express XT-I and XT-II from patients with AF before [1, 8, 44]. For the expression analyses in the present project, we divided the tissues in four categories: vascular tissue (V), synovial tissue (S), hypocellular fibrosis (HYP-F), and hypercellular fibrosis (HYPR-F). Compared to the current controls, xylosyltransferase expression was increased as far as the synovialis in conjunction with hypercellular fibrosis, and the order of XT-I and XT-II expression in the tissue sequence below in AF patients was noted:

$$ V\kern0.48em >>\kern0.36em HYPR-F> HYP-F>S $$

The AF patients’ vessels revealed at least twice as high an expression of XT-I as in any of the other tissues types. The control specimens exhibited similar XT-I expression in HYP-F and HYPR-F, while their vessels expressed just minimally elevated XT-I. Higher XT-I values were observed in the synovialis. XT-II expression in the various tissues resembles that of XT-I, but it tends to be much less.

The myoblast population also seems to originate in various cell types in conjunction with AF. The strongly expressed XT-I in vessels of AF patients seems to argue for the existence of resident perivascular cells that resemble MSC, which provide a source for the formation of myofibroblasts in AF, as in other types in fibrosis. It has been shown that MSC-like cells participate in the formation of kidney fibrosis [30, 45]. Recent research has provided evidence that specific subsets of tissue-resident mesenchymal cells are the major source for injury-induced matrix-producing fibroblasts and myofibroblasts in multiple organs [26]. At the steady state, the perivascular niche contains different subsets of MSCs. After an injury, resident perivascular MSC-like cells are activated around vessels, a process leading to their detachment from the vessel wall and to differentiation into ECM-producing myofibroblasts [26]. Cells of this phenotype are regulating scar tissue formation and immune cells’ recruitment and their activity. ECM, chemokines, and growth factors produced by damaged epithelial cells, endothelial cells, and inflammatory cells also play a role in this process. Failure to terminate this repair program leads to fibrosis by hyperactivity of fibroblasts. It is also possible that myofibroblast progenitors are also localized in the perivascular niche [26]. Even though MSCs can be generated in vitro from pericytes or adventitial cells, their precise identity and function in vivo remain unclear and will require further investigation.

Resistant MSC-like cells also play a role in the development of pulmonary fibroses [25], cardiac fibroses [46], liver fibroses [47], dermatological fibroses [48], and bone marrow fibroses [49].

In the fibrotic connective tissue of AF patients (HYP-F and HYPR-F), myofibroblasts develop out of fibroblasts [10, 50] and out of synoviocytes in their synovialis [51].

XT-I und XT-II exhibit similar structures [34]. Both are capable of catalyzing the posttranslational xylosylization of proteoglycan core proteins. Assessments of XT activity in serum revealed no differences in serum XT-I activity between AF patients and controls; however, serum XT-II activity dropped as the fibrosis became more severe [10, 52].

The main limitation of the present study is the low number of tissue samples for access to XT analysis. Another limitation is that we do not know the disease status of the control group. Since the expression of XT in the control group was rather low, it can be assumed that the human cadavers did not exhibit arthrofibrosis of the knee joint and are therefore suitable as a control group.

There is no data reported in the literature on what constitutes systematic and specific criteria for diagnosing histopathologically an AF. Providing evidence of increased XT activity could eventually make a histopathological diagnosis in connection with clinical evidence possible. Measuring XT activity in a biopsy of arthrofibrosis patients would be helpful, but the high risk of infection after puncture of the knee joint should be taken into account. Further investigations are necessary to define criteria for AF, similar to the criteria which are found for β catenin [20] and to prove the participation of various cell types in the development of AF. The current treatment of arthrofibrosis involves surgery. It would be more effective to treat the dysregulation of myofibroblasts with the help of pharmaceuticals. Therefore, it is very important to know and understand the pathogenesis of arthrofibrosis. An effective therapeutic approach may involve regulating cytokines and mediators like TGF-β, Il-, or TNF alpha antibodies. More studies are required to prove the participation of various cell types in AF’s development, as only then it will be possible to develop successful therapies selectively applying anti-fibrotic agents.

## Conclusion

The current study shows that strongly increased myofibroblastic differentiation is accompanied by a highly elevated XT-I rate of synthesis and a much lower XT-II synthesis rate.

## Data Availability

The datasets used and/or analyzed during the current study are available from the corresponding author on reasonable request.

## Abbreviations

ACL: Anterior cruciate ligament
AF: Arthrofibrosis
ECM: Extracellular matrix
HE: Hematoxylin/eosin
HYP-F: Hypocellular fibrosis
HYPR-F: Hypercellular fibrosis
IRS: Immunoreactive scoring system
PDGF: Platelet-derived growth factor
ROIs: Regions of interest
S: Synovial tissue
TGF-β1: Transforming growth factor β1
TKA: Total knee arthroplasty
V: Vascular tissue
XT: Xylosyltransferases
